# Laser Treatment of Surfaces for Pool Boiling Heat Transfer Enhancement

**DOI:** 10.3390/ma16041365

**Published:** 2023-02-06

**Authors:** Łukasz J. Orman, Norbert Radek, Jacek Pietraszek, Janusz Wojtkowiak, Marcin Szczepaniak

**Affiliations:** 1 Faculty of Environmental, Geomatic and Energy Engineering, Kielce University of Technology, Al. Tysiaclecia P.P. 7, 25-314 Kielce, Poland; 2 Faculty of Mechatronics and Mechanical Engineering, Kielce University of Technology, Al. Tysiaclecia P.P. 7, 25-314 Kielce, Poland; norrad@tu.kielce.pl; 3 Faculty of Mechanical Engineering, Cracow University of Technology, Al. Jana Pawła II 37, 31-864 Cracow, Poland; jacek.pietraszek@mech.pk.edu.pl; 4 Institute of Environmental Engineering and Building Installations, Poznan University of Technology, Pl. M. Skłodowskiej-Curie 5, 60-965 Poznan, Poland; janusz.wojtkowiak@put.poznan.pl; 5 Military Institute of Engineer Technology, Ul. Obornicka 136, 50-961 Wroclaw, Poland; szczepaniak@witi.wroc.pl

**Keywords:** boiling, heat transfer enhancement, laser beam, surface treatment

## Abstract

The laser treatment of surfaces enables the alteration of their morphology and makes them suitable for various applications. This paper discusses the use of a laser beam to develop surface features that enhance pool boiling heat transfer. Two types of structures (in the ‘macro’ and ‘micro’ scale) were created on the samples: microfins (grooves) and surface roughness. The impact of the pulse duration and scanning velocity on the height of the microfins and surface roughness at the bottom of the grooves was analyzed with a high precision optical profilometer and microscope. The results indicated that the highest microfins and surface roughness were obtained with a pulse duration of 250 ns and scanning velocity of 200 mm/s. In addition, the influence of the ‘macro’ and ‘micro’ scale modifications on the boiling heat transfer of distilled water and ethyl alcohol was studied on horizontal samples heated with an electric heater. The largest enhancement was obtained for the highest microfins and roughest surfaces, especially at small superheats. Heat flux dissipated from the samples containing microfins of 0.4 mm height was, maximally, over three times (for water) and two times (for ethanol) higher than for the samples with smaller microfins (0.2 mm high). Thus, a modification of a selected model of boiling heat transfer was developed so that it would be applicable to laser-processed surfaces. The correlation proved to be quite successful, with almost all experimental data falling within the ±100% agreement bands.

## 1. Introduction

Laser technology has a broad range of applications, especially in the area of surface engineering. It enables, among other things, the design and alteration of surface morphology on both micro and macro scales. Thus, it can also be effectively used for the production of highly efficient heat exchangers, especially for phase-change heat transfers such as boiling and condensation. These modes of heat transfer are utilized, for example, in refrigeration and heat pumps, as well as other devices (e.g., for waste energy recovery), and are characterized by considerable heat fluxes that can be exchanged at small temperature differences. 

Boiling involves both a change of phase from liquid to vapor and the convective movement of bubbles, thus, it is a highly effective mode of heat transfer. Additional improvement in the amount of heat (heat flux) dissipated from a surface can be obtained if the surface is extended or roughened. Papers [1,2,3] dealing with pool boiling on extended surfaces, in the form of fins produced with machining techniques, indicate a considerable improvement in heat transfer when compared to a smooth surface. Similarly, studies dealing with boiling on rough surfaces generated with emery paper and other mechanical methods (e.g., [4,5,6]) prove that such rough heat exchangers dissipate higher heat fluxes in relation to a smooth reference surface. Consequently, the combination of both of these methods can be especially advantageous. In practice, this can be achieved with the laser technique. The laser beam modifies the surface morphology on the macro scale producing pre-designed shapes on the surface (e.g., in the form of longitudinal fins) as well as on the micro scale through the generation of a rough surface due to the melting and evaporation of the material. Such dual surface modification is the focus of the present paper.

The application of laser treatment for pool boiling heat transfer enhancement is relatively new; however, the existing literature data generally indicate the highly advantageous influence of this surface modification technique on the thermal performance of heat exchangers. Kruse et al. [7] studied water pool boiling on stainless steel surfaces modified with a femtosecond laser. The coatings contained mound-like microstructures covered by dense layers of nanoparticles. It was reported that the maximal heat transfer coefficient was almost three times higher compared to the reference (polished) sample. Experiments performed by Ho et al. [8] by the boiling of HFE-7000 on surfaces with microstructures made of AlSi10Mg powder, via selective laser melting that produced microcavities and microgrooves, indicated over 30% heat transfer enhancement compared to a smooth surface. A later study by these authors [9] focused on FC-72 boiling on micro-cavities as well as microfin heaters produced with a laser using AlSi10Mg base powder. The average heat transfer coefficient rose by about 70%, while the critical heat flux rose by about 76% relative to the reference sample. It was also noticed that microfins were more efficient than micro-cavities. In both papers by these same authors [8,9], a modified Rohsenow correlation (which was originally developed for smooth surfaces) was proposed and compared with the experimental results. The FC-72 boiling agent was also used by Liu et al. [10] who analyzed laser-treated surfaces with various spacings as well as peak-to-valley heights. The authors reported a large enhancement in the heat transfer coefficient; it was almost six times higher compared to the smooth surface and had an over 90% improvement in critical heat flux. Voglar et al. [11] experimentally studied water boiling on thin stainless-steel foils treated with a nanosecond fiber laser. The tests covered three types of patterns (parallel lines, squares, and circles) generated on the specimens. All the modified surfaces improved the boiling conditions, with the coefficient of heat transfer being over four times higher in comparison to a plain surface. The same base material in the form of thin foils was applied by Zakšek et al. [12] to analyze the boiling of water, ethanol, and their mixtures. The laser beam used generated lines of various widths (from 0.5 to 2.5 mm). All of these laser-treated surfaces enhanced boiling, with the largest improvement of the heat transfer coefficient reaching almost three times the value for the smooth surface. The authors indicated that laser-generated microcavities reduce the temperature of the onset of boiling as well as increase the density of nucleation sites (locations where vapor bubbles are developed) and nucleation frequency. A significant rise in active nucleation site density (by twenty to forty times) and the heat transfer coefficient (110% larger compared to the smooth surface) was also reported in a study [13] on boiling. The experiments took place on steel foils covered with laser-made lines. Zupančič et al. [14] investigated boiling on polydimethylsiloxane-silica coatings treated with a nanosecond laser. The authors reported that almost all the samples generated smaller bubbles and showed a higher nucleation frequency and larger density of active nucleation sites in relation to the plain surface. The performance of only one surface (containing shallow microstructures without any cavities) was comparable to that of the untreated sample. The same kind of coating base was also modified with a laser in [15] to produce square patterns of 0.25, 1, and 2 mm^2^ spot sizes. The specimen with the 0.25 m^2^ spot size dissipated the highest heat fluxes and the performances of the other two were similar to one another. Sitar et al. [16] examined surfaces textured with a laser and found that their boiling performance was generally better than the smooth surface (although some samples at certain superheated temperatures actually performed worse). Može et al. [17] observed that the heat transfer coefficient was over 115% larger for the laser-textured samples compared to the smooth surface sample. Moreover, these textured surfaces proved to provide more stable boiling conditions. Grabas [18] tested the boiling of water on steel heaters and recorded a considerable improvement for laser-vibration textured surfaces, with the heat transfer coefficient being elevated over four times. A combined effect of the application of lasers and the use of ethanol-based nanofluids was investigated by Karthikeyan et al. [19]. An almost 60% boiling heat transfer enhancement, in comparison to the smooth surface and pure base liquid, was reported. Dharmendra et al. [20] analyzed water boiling on three copper surfaces containing laser-made square grooved patterns of different depths (30, 70, and 100 μm). The deepest grooves proved to be most advantageous, but all the samples improved boiling conditions, especially for small superheats. Surfaces with longitudinal grooves generated with a nanosecond laser were tested by Nirgude and Sahu [21] with acetone as the boiling agent. All the samples provided enhanced heat transfer conditions. The authors also compared their test results with the Rohsenow correlation. Microstructures in the form of microfins were investigated by Orzechowski [22] for FC-72 boiling, however, the samples were non-isothermally heated. The laser treatment provided augmentation of the heat transfer in relation to the smooth surface—mostly at low heat fluxes. At higher ones, the performance of the treated and untreated samples was quite similar. 

Laser treatment can also produce porous structures on the heat exchanging surfaces. Wang and Leong [23] analyzed the boiling of FC-72 on a layer made with the selective laser melting method using AlSi10Mg powder shaped to form octet-truss unit cells of heights 2.5, 5, and 10 mm. The mean heat transfer coefficient turned out to be over 2.8 times higher than that for a smooth surface. The same production method was used by Zhang et al. [24] to generate 3D grid structures. The authors discovered that not all the specimens improved heat transfer. A later paper by Zhang et al. [25] also focused on a 3D structure, however, it was made of shells and an internal open channel. The authors concluded that all the samples improved the heat transfer performance in relation to a plain surface. Serdyukov et al. [26] modified a silicon surface with a Nd:YAG laser and recorded almost a 50% heat flux enhancement compared to the reference sample for water boiling. Moreover, the authors reported that laser treatment increased nucleation site density and nucleation frequency (the frequency rose about 15 times), but bubble departure diameters decreased (on average, over five times)—all in relation to the reference silicon surface. Very recently, Eid et al. [27] experimentally analyzed boiling on a surface covered with micro-cavities generated with a fiber laser. Three shapes of cavities were tested: cylindrical, cubic, and pentagonal—all with a depth of 500 μm. The pentagonal cavities proved to be the most effective, with a maximal enhancement of heat flux over 120% for water. Zupančič et al. [28] studied the surface phenomena during water boiling on thin foils treated with a nanosecond fiber laser to form triangular lattice patterns. The surface with the highest density of cavities performed the best. All the studied surfaces (whose nucleation site density varied from 9 to 51 sites/cm^2^) showed very similar bubble footprint radiuses, growth times, and wait times as well as nucleation temperatures. The authors proposed a semi-empirical correlation for the heat transfer coefficient but stated that it was not meant to be a generalized correlation.

Laser textured heaters also enhance flow boiling heat transfer, as described by Piasecka et al. [29] who tested vibration-assisted laser-treated surfaces. The local heat transfer coefficient for the saturated boiling region was found to be considerably higher than for the smooth surface at the same heat flux. In the flow boiling mode of heat transfer, surface phenomena and other methods of surface modifications can also play a significant role, as pointed out, e.g., in [30,31,32,33].

The technology of generating coatings with a laser is generally known but is mostly focused on tribological properties. Vilhena et al. [34] analyzed the action of a pulsed Nd:YAG laser on steel and the micropores generated as a result. (The pulse energy varied from 0.5 to 8.3 mJ, with a pulse duration of 98–597 ns.) It was observed that the pore diameter generally increased with rising pulse energy, while the depth of the pores became smaller as the energy rose. In multimode action, a larger number of pulses led to cupped and smoother pores while the pore diameter remained steady. Zuhlke et al. [35] analyzed the creation of micro- and nanostructured surfaces on nickel substrates through above-surface growth and below-surface growth mechanisms with the use of a femtosecond laser. It was reported that growth mechanisms include, among others, the ablation of surface valleys, the flow of melt, and the redeposition of the ablated material. 

It needs to be noted that some of the papers [34,35] were aimed at tribological applications and not heat transfer properties. The research works considering the influence of laser treatment on pool boiling are quite new, very rare, and cover only a few samples’ experiments. The first to mention pool boiling were Nirgude and Sahu [36], who investigated the influence of laser processing parameters on water pool boiling performance with six samples. A nanosecond laser was used in the study and samples were treated with varying wavelengths of 1064 nm, 355 nm, and 532 nm (all at 10 Hz) and with laser powers from 0.8 W to 4.6 W; the surface roughness produced ranged from 0.237 to 0.496 μm. A significant leftward shift in the boiling curves of the laser-treated surfaces was observed. The highest enhancement of boiling was reported for the biggest wavelength (and highest laser power). It was concluded that microstructure formation due to laser action was the prime reason for heat transfer improvement. However, they also stated that the exact mechanisms behind the creation of the organized surface microstructures are not known. The paper does not provide a comprehensive study due to its small number of samples and varying parameters. The other paper by Nirgude and Sahu [37] compared the water pool boiling performance of samples produced with two laser systems: the pulsed Nd:YAG and continuous wave laser systems. Six samples were produced, all with a wavelength of 1064 μm. The laser power was 4.0–4.6 W for the pulsed laser and 40–50 W for the continuous one. The maximal enhancement (over two times) was recorded for the continuous wave laser and the largest laser power (50 W). The presence of grooves, pores, and microcavities was stated as the reason for the enhancement. It needs to be noted, however, that the grooves were too small to consider them as ‘microfins’, which is the focus of the present paper.

As can be concluded, boiling heat transfer studies on laser-processed surfaces are typically focused only on the experimental analysis of the thermal performance of the produced samples. Although in some of the above-mentioned papers, the test results were compared with the Rohsenow correlation (which was actually developed for smooth surfaces about seven decades ago) or with a proposed semi-empirical correlation, there was no development of a new model or correlation for boiling heat flux determination on laser-treated surfaces. Thus, based on the literature review, it can be stated that although laser-treated heaters were tested by other authors under pool boiling conditions, no paper, to the best of our knowledge, has been found that provides a thorough analysis of the impact of laser beam technology on the development of a pre-designed heat enhancing (in the ‘micro’ and ‘macro’ scale) surface morphology, combined with the heat exchange characteristics of such surfaces, together with the boiling model development. Consequently, this is the motivation and focus of the current study.

It also needs to be emphasized that the authors of the above-mentioned papers on boiling on laser-treated surfaces have not considered the issue of boiling enhancement from the point of view of dual surface modification with the laser beam, namely, the longitudinal fins (‘macro’ scale) on the surface and the generation of a high roughness surface area between the fins (‘micro’ scale). Such dual surface modification is the issue considered in the present paper.

The laser treatment can significantly improve boiling performance; however, some literature reports seem to question that concept, at least for some ranges of heat flux values. The present paper also aims to clarify this issue with experimental data of the pool boiling of water and ethanol as well as the modification of a selected correlation for boiling heat flux. It also needs to be emphasized that a modification of the Xin and Chao model with the view to apply it to laser-processed surfaces has never been accomplished before and will be presented in the current paper. 

The development of efficient heat exchangers is currently an important engineering task, as pointed out in very recent papers by Križo et al. [38] and Červenka et al. [39]. Consequently, the study has also a practical aspect of providing data for the development of efficient heat exchangers with the use of highly precise, efficient, and reliable laser technology. Such heat exchangers can be made from surfaces that have been proven to generate the highest thermal performance. Moreover, other laser-treated surfaces can be designed based on the conclusions derived from the experimental results and the modified correlation obtained in the current study.

## 2. Materials and Methods

**Figure 1:**
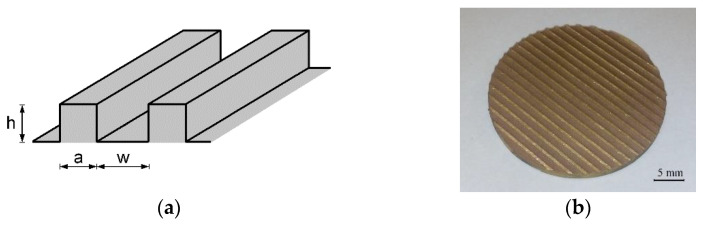
A selected laser-treated sample: (**a**) schematic drawing and (**b**) photo.

The study was performed with distilled water and ethyl alcohol (of purity 99.8%) as the boiling agents. The specimens, on which the boiling phenomenon occurred, had the form of copper discs 3 cm in diameter. The SPI G3.1 SP20P pulsed fiber laser was used to generate longitudinal grooves on the whole surface. Figure 1 presents the schematic (a) and photo (b) of the grooved sample. 

**Figure 2:**
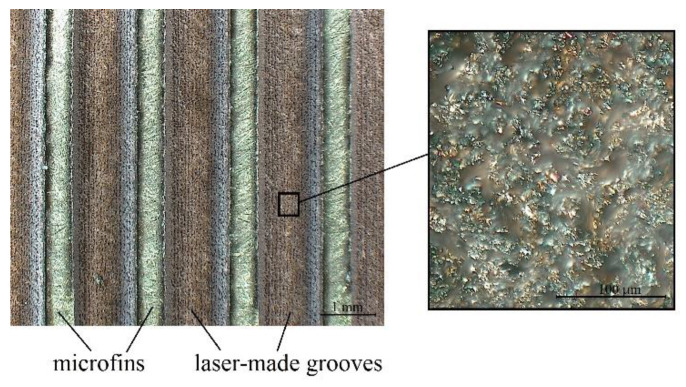
The surface morphology details (mag. 35×, left-hand side picture), with the bottom microstructure (mag. 700×, right-hand side picture).

The magnified image of the parallel grooves and the bottom microstructure is shown in Figure 2.

The laser treatment required a precise selection of process parameters so that the material could be shaped into pre-designed patterns [40,41]. The following process parameters were set in the laser system control unit for the generation of the laser-treated surfaces in the present study:

- Impulse frequency: 25, 60, and 120 kHz;
- Pulse duration: 60, 120, and 250 ns;
- Scanning velocity: 200 and 400 mm/s;
- Focal spot size: 35 μm,
- Power: 20 W.

The specimens differed by the values of depth (h) and width (w) of the grooves as well as the microfins’ width (a) (as presented in Figure 1a). Table 1 lists the geometrical parameters of the circular samples tested in the study and used for the development of the correlation (modification of the boiling model).

Moreover, the tests of the influence of the process parameters on the sample microgeometry were conducted on a copper flat bar (the same material as the circular samples for boiling heat transfer investigations). Here, the obtained depths of the grooves ranged from 0.24 to 0.89 mm, while surface roughness was from 5.27–9.60 μm. 

**Figure 3:**
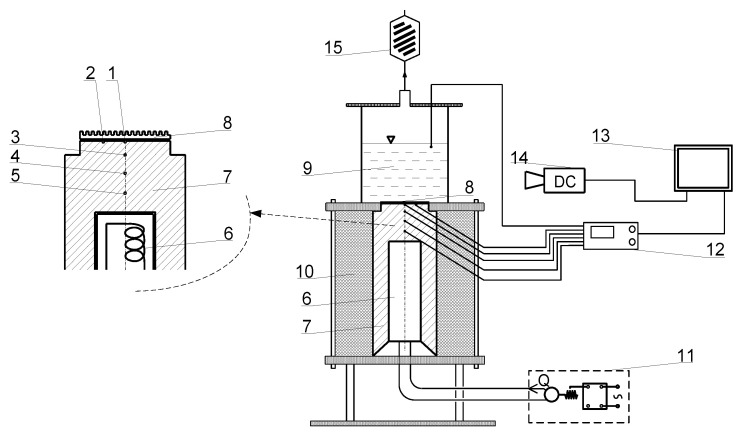
Boiling test set-up: 1 and 2, thermocouples below specimen; 3, 4, and 5, thermocouples in the copper block; 6, heater; 7, copper block; 8, specimen; 9, fluid; 10, thermal insulation; 11, power supply; 12, temperature recording unit, 13, computer; 14, digital camera; and 15, water-cooled coil (figure based on the authors’ work [42]).

The analysis of the microstructure of the laser-textured surfaces was conducted on two high-precision optical devices with adequate data treatment software (the optical profilometer Talysurf CCI equipped with the TalyMap Platinium software as well as Hirox KH-8700 optical microscope), while the boiling tests were performed on the experimental stand described in detail by Orman et al. [42], together with error analysis and calibration data (as an open access paper). The main element of the experimental set-up is the copper heating block with an electric resistance heater inside it, on which laser-treated samples were soldiered. Above the sample, a glass vessel with a boiling agent (water or ethanol) is located (Figure 3). It rests on the Teflon plate, in which a close-fit hollow for the sample was made. The heat conducted on the sample increases its temperature over the saturation temperature of the liquid and boiling occurs. Generated vapor is condensed and returned to the vessel so that the level of the liquid is constant and maintained throughout the experiments. Condensation of the vapor occurs within the cooling coils supplied with cold water. The experiments were conducted under ambient pressure. The temperature values within the block were determined with K-type thermocouples, connected to the data logger Keithley 2700 with the multi-thermocouple extension set. The regulation of the heat flux was achieved with an autotransformer and the rising steps of the voltage enable the generation of increased superheated values. Thus, the dependence between the superheat and the heat flux can be obtained in the form of so-called ‘boiling curves’ and subsequently analyzed for each sample to determine its boiling performance and enhancement potential. 

The thermal performance of each sample was determined as a function of the superheat (θ), which is the difference between the sample surface temperature and the saturation temperature vs. heat flux (q) dissipated to the liquid. The heat flux was calculated using Fourier’s law of conduction, taking into account the temperature gradient within the copper block according to the following equation:

(1) $$q=\frac{\lambda_{\mathrm{Cu}}}{L_{5-3}}\left(T_{5}-T_{3}\right),$$

**Figure 4:**
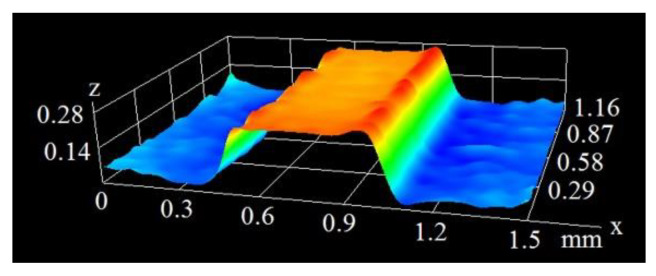
Image of a microfin from a pulse duration of 60 ns and velocity of 400 mm/s.

In the above equation, T_3,_ and T_5_ are thermocouple temperature readings at locations in Figure 4 and L_5−3_ is the distance between thermocouples 5 and 3 (l_5−3_ = 15.97 mm). For verification purposes, heat flux was also calculated between thermocouples 5 and 4 as well as 4 and 3 using the same formula.

The temperature at the heater surface (on which the laser-textured samples are soldered) was determined from the extrapolation of the mean temperature value under the sample:

(2) $$T=T_{1,2}-\frac{{\mathrm{qL}}_{a}}{\lambda_{\mathrm{Cu}}},$$

where L_a_ describes the distance between the axis of thermocouples 1 and 2 (as in Figure 4) and the heater surface (L_a_ = 3.25 mm) and T_1,2_ is the mean value of the temperature readings of thermocouples 1 and 2 located under the considered sample. As mentioned earlier, the wall superheat is the difference between the surface temperature and the saturation temperature:

(3) $$\theta =T-T_{\mathrm{sat}},$$

It needs to be noted that the heat flux determination method used in the study is proven and commonly applied in pool boiling tests, e.g., in [1,2,3,18]. High-speed visual studies of boiling on microfins are also conducted with similar equipment and methods (e.g., [1,2,3]). The accuracy analysis revealed that the highest experimental uncertainties occur in the region of low heat fluxes and become lower for the highest heat flux values (reaching about 4%), while the calibration data indicated that the results for the smooth reference sample obtained at the experimental stand are within those reported in the literature (as presented in [42]). A high-speed digital camera by PhotonFocus with an LED lighting set (located around the lens) was used in the study to observe the behavior of vapor bubbles generated on the laser-textured samples. The lens was situated about 5 cm from the sample on which the boiling process occurred, and the focus was set manually. The maximal sequence recording frequency was set in the software of the camera at 488 frames per second.

## 3. Results and Discussion

### 3.1. Laser Processing

The impact of the laser processing of surfaces used as phase-change heat exchangers can be considered in the “macro” scale as a surface extension (in the form of microfins or cavities) and “micro” scale (in the form of increased roughness caused by the laser beam which increases nucleation sites’ density). Both of these factors have a considerable effect on boiling heat transfer enhancement and will be considered in the present study.

**Figure 5:**
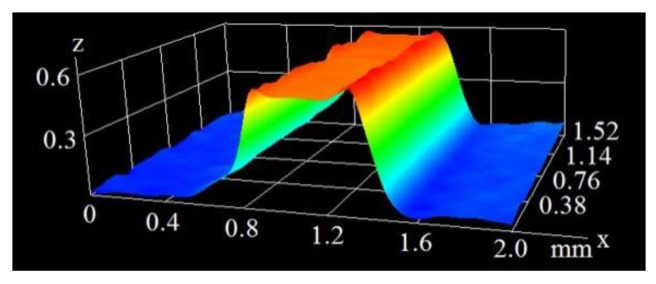
The image of a microfin from a pulse duration of 60 ns and velocity of 200 mm/s.

The extension of the surface with the creation of microfins (as presented in Figure 1) can be achieved with various techniques, such as mechanical milling, however, the laser beam action provides a more favorable shape of the fin, with a width that becomes thinner at the top. This makes the vapor removal from the heating surface easier and the heat exchanger more efficient. The process of the creation of microfins with the laser beam is possible due to the evaporation of the material at the site of the beam’s interaction with the surface. Figure 4 and Figure 5 present the optical microscope images (in pseudocolor) of microfins generated on the copper base at the same pulse duration of 60 ns and different scanning velocities of 200 and 400 mm/s.

**Figure 6:**
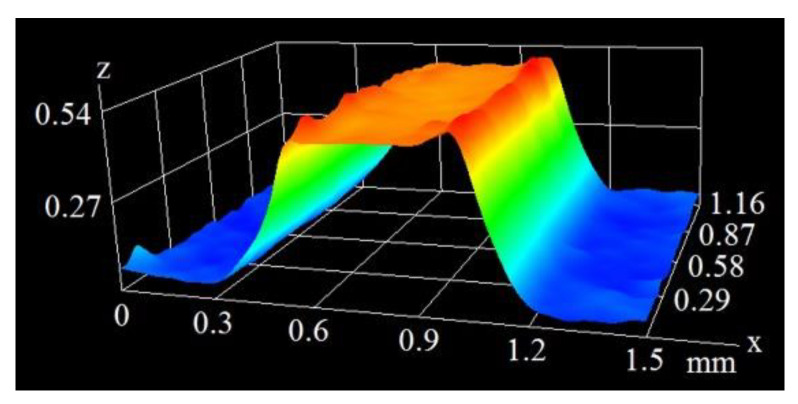
The image of a microfin from a pulse duration of 120 ns and velocity of 400 mm/s.

**Figure 7:**
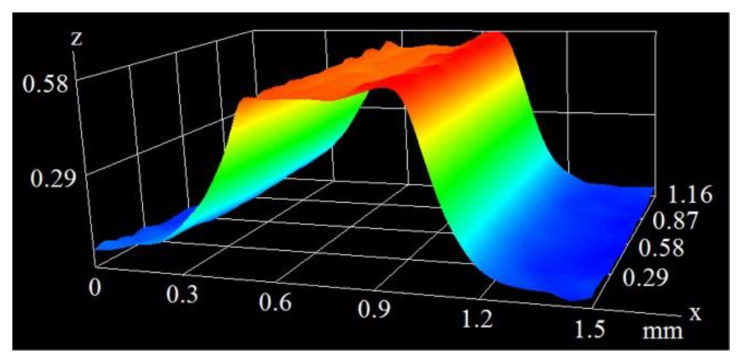
The image of a microfin from a pulse duration of 250 ns and velocity of 400 mm/s.

As can be seen in the above figures, the mean height of the microfins generated at the 200 mm/s scanning velocity was 0.545 mm. At a velocity of 400 mm/s, this value was much smaller and amounted to 0.237 mm. Figure 6 and Figure 7 show the microscopic images of the microfins produced at the same velocity of 400 mm/s (as in Figure 4), but with different pulse durations of 120 and 250 ns. 

Figure 6 and Figure 7, combined with data in Figure 4, clearly show that as the pulse duration increased from 60 ns, through 120 ns to 250 ns, the height of the microfins (namely, the depth of the grooves generated on the surface) rose from 0.237 mm through 0.466 mm to 0.642 mm. This is because when the interaction of the laser beam with a given spot on the surface lasts longer, the amount of material undergoing phase-change increases, and the depth becomes larger.

**Figure 8:**
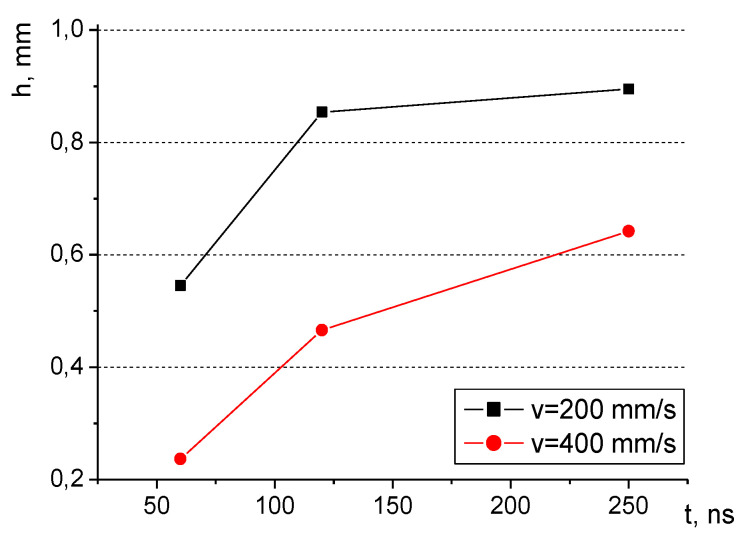
Relationship between pulse duration and groove depth for two scanning velocities.

However, the relation between the pulse duration (t) and the obtained groove depth (h) for both scanning velocity (v) values is not linear, as presented in Figure 8; the curves seem to flatten as pulse duration increases. It seems that as the duration rises, the ease at which the material evaporates diminishes and it becomes more difficult as time passes. The phenomenon can be observed for both considered velocity values but is more pronounced for the smaller velocity due to the fact that the velocity itself has an impact on groove depth. For smaller velocities, the interaction with the material lasts longer and a larger amount of the surface material can evaporate. Thus, it magnifies the influence of the pulse duration and confirms the findings presented in Figure 8.

The extension of the surface in the macroscale (for example in the form of microfins) is vital for boiling heat transfer, however, it seems equally important to investigate the roughness of the surface below the laser beam (namely, at the bottom of the grooves) due to the fact that increased roughness is also a factor that favorably influences the boiling heat transfer performance.

**Figure 9:**
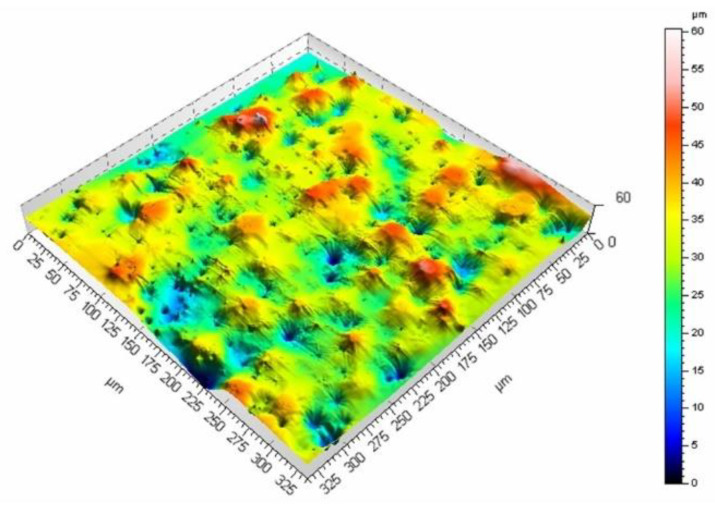
Surface morphology produced from a pulse duration of 60 ns and velocity of 400 mm/s.

**Figure 10:**
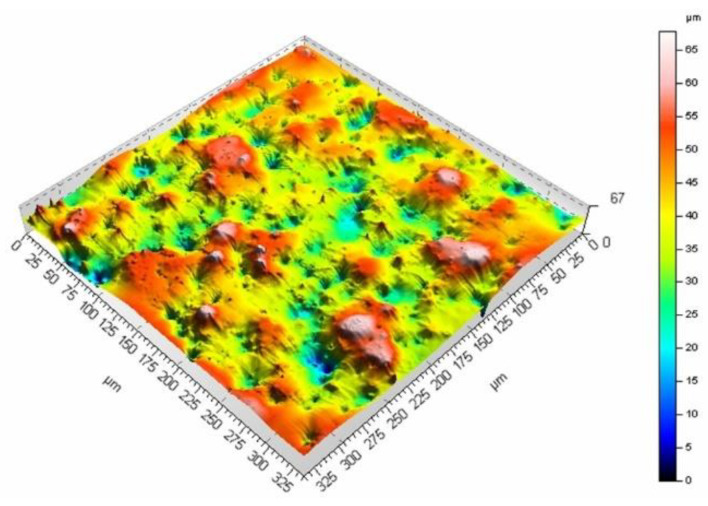
Surface morphology produced from a pulse duration of 60 ns and velocity of 200 mm/s.

The surface roughness generated with the laser treatment is dependent on the process parameters. The investigation of this phenomenon was focused on the analysis of the surface morphology produced with two scanning velocities and three pulse duration values: 60 ns, 120 ns, and 250 ns. Figure 9 and Figure 10 show the surface morphology for two scanning velocities of 200 and 400 mm/s and a pulse duration of 60 ns.

**Figure 11:**
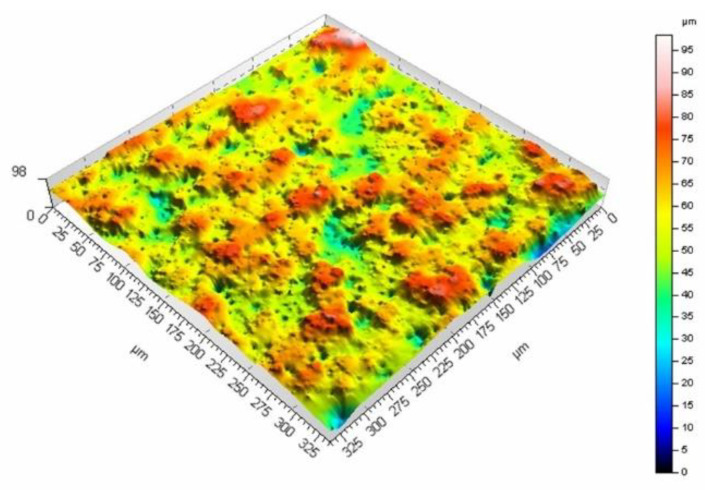
Surface morphology produced from a pulse duration of 120 ns and velocity of 200 mm/s.

**Figure 12:**
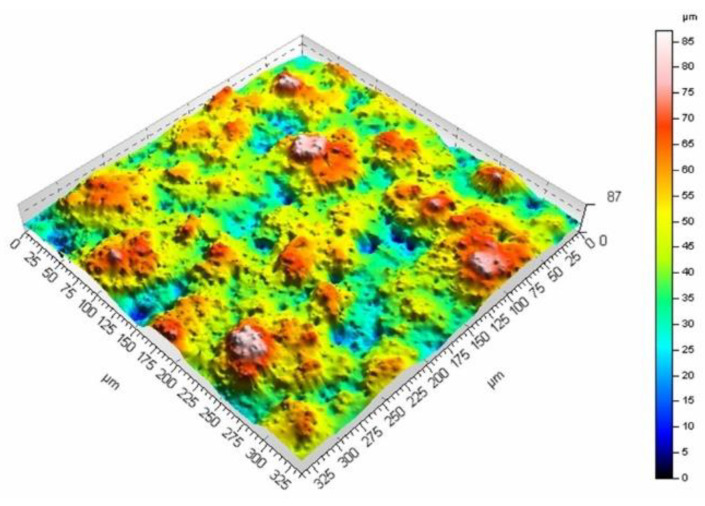
Surface morphology produced from a pulse duration of 250 ns and velocity of 200 mm/s.

The mean surface roughness of the sample treated with the laser beam at the scanning velocity of 200 mm/s amounted to 6.98 μm, while for the velocity of 400 mm/s, it was 5.27 μm. The surface processed at a lower velocity is rougher (as evidenced by the mean surface roughness and Figure 9 and Figure 10). A smaller velocity, and thus, the longer interaction of the laser beam with the surface seems to produce deeper and higher surface elements, which leads to larger mean surface roughness values. The same phenomenon can be observed if the pulse duration is increased while maintaining the same scanning velocity. Figure 11 and Figure 12 present the surface morphology of samples that underwent laser processing at a scanning velocity of 200 mm/s and pulse duration values of 120 ns and 250 ns. 

**Figure 13:**
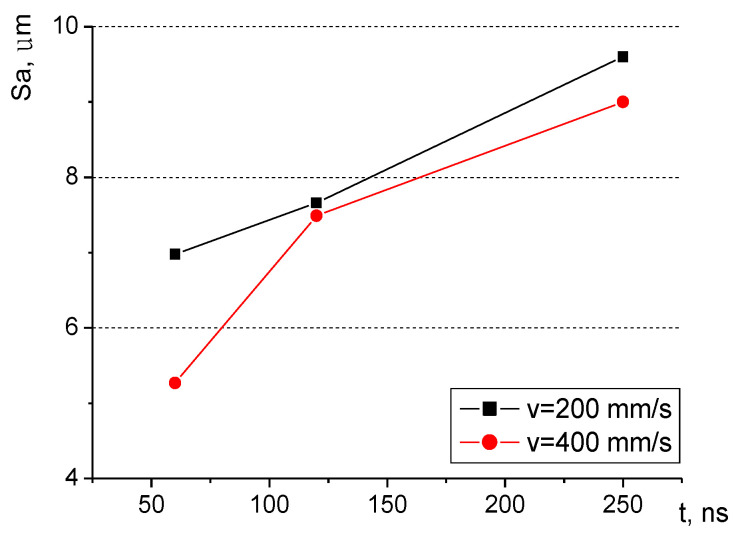
Relationship between pulse duration and surface roughness for two scanning velocities.

The results (together with the data in Figure 10 for the pulse duration of 60 ns) confirm the influence of the pulse duration on surface roughness, with values that amounted to 7.66 μm (for the 120 ns duration) and 9.6 μm (for the duration of 250 ns). Figure 13 presents the dependence of surface roughness on the pulse duration values for two scanning velocities.

The surface of the laser-treated area becomes rougher mostly with the increase in the pulse duration value, however, the influence of the scanning velocity can also be quite visible, especially for a very low pulse duration (namely, 60 ns). 

**Figure 14:**
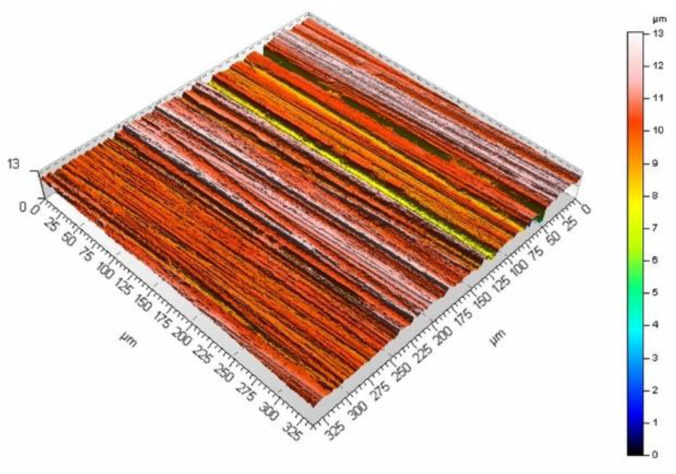
The surface morphology of a surface treated with emery paper no. 240 (mean roughness: Sa = 0.79 μm).

It needs to be noted that in the case of boiling heat transfer, the impact of surface microgeometry is considerable. Typically, the tests found in the literature on the influence of roughness on boiling heat flux were performed on samples treated with emery paper, however, such surfaces are characterized by a very different morphology (as evidenced in Figure 14) than that obtained with laser processing. Thus, the results given in the literature for heaters treated with emery paper cannot relate directly to those obtained as a consequence of laser treatment. 

**Figure 15:**
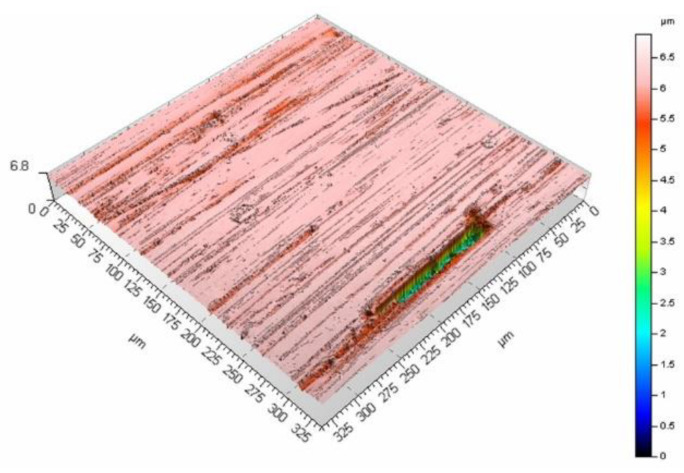
The surface morphology of a smooth surface example (mean roughness: Sa = 0.17 μm).

The results of boiling heat transfer presented in the next section will be compared with the data for a smooth reference surface. Figure 15 presents an example of smooth surface morphology. Its surface roughness is over one order of magnitude lower than that generated during laser processing and amounted to 0.171 μm (with a standard deviation of 0.198 μm). It might also contain some scratches on the surface, but they are not common and are typically shallow. The production technology consisted of mechanical milling (thus, the presence of scratches) and fine smoothing afterward for a proper surface finish. 

### 3.2. Pool Boiling Heat Transfer Analysis 

**Figure 16:**
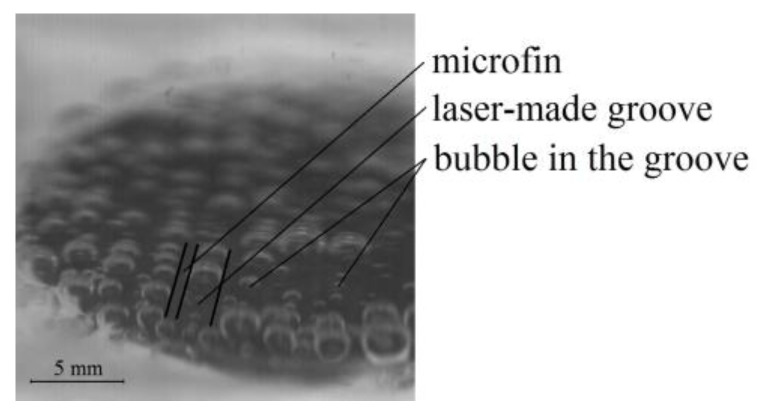
Vapor bubbles on a laser-treated surface.

Nucleate boiling is a highly intensive process of heat transfer in which an important element is the growth and departure of vapor bubbles. Their movement enhances convection and carries away vapor. Thus, the heat fluxes exchanged at boiling can be considerable. The bubbles grow at locations called nucleation sites, which are often cavities, micro-scratches, and other irregularities on the surface. Consequently, increased roughness can favorably impact the boiling performance. Figure 16 confirms this claim. It presents the laser-processed sample with a 0.4 mm groove depth and 1.5 mm groove width. The vapor bubbles line up along the microfins and are typically present at the bottom of the laser-made grooves due to their increased roughness. 

**Figure 17:**
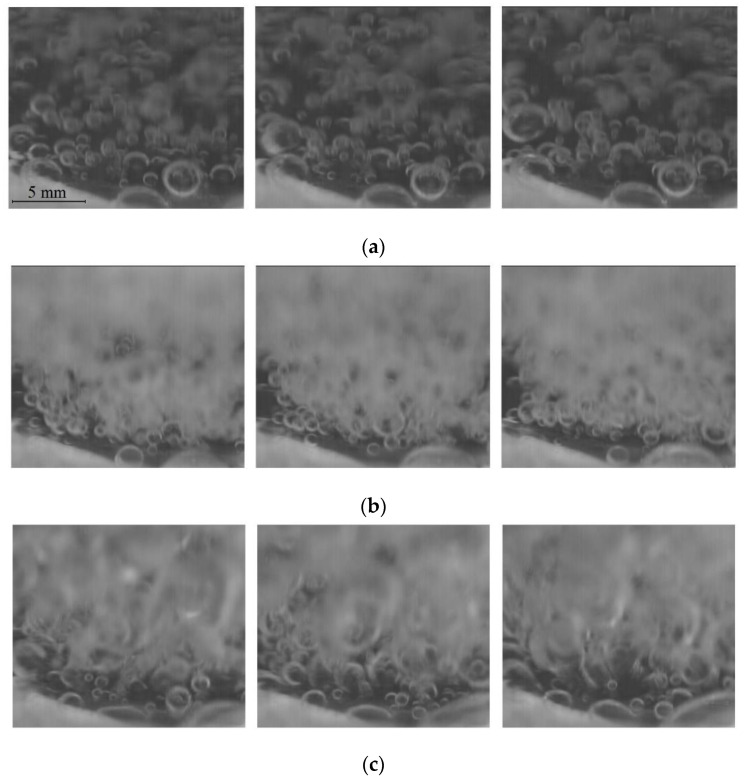
Boiling performance of a sample with a 0.4 mm groove depth and 1.5 mm groove width at a (**a**) heat flux of 20.7 kW/m^2^; (**b**) heat flux of 45.1 kW/m^2^; and (**c**) heat flux of 88.2 kW/m^2^.

The above picture was recorded at a very low heat flux value. Bubbles can also form at the tips of the microfins if the temperature from high heat fluxes is large enough there. Figure 17 presents a sequence of photographs taken with a high-frequency digital camera with specially designed LED lighting for three heat flux values and the sample as seen in Figure 16. 

It seems that the largest impact of surface roughness might be observed for small superheats when bubbles typically grow within the grooves on the rough surface. As the heat flux increases, this impact should diminish (as confirmed later with the experimental heat flux data). It is also in line with a generally accepted notion that the superheat needed to initiate boiling depends on the size of the nucleation site. The boiling process can begin at low superheats if the size of the nucleation site is small enough (for example, a scratch on the surface). Thus, rough surfaces that possess large numbers of irregularities effectively promote boiling at low-temperature differences leading to enhanced heat transfer in this range of superheated temperatures. At higher temperature values this favorable phenomenon diminishes.

**Figure 18:**
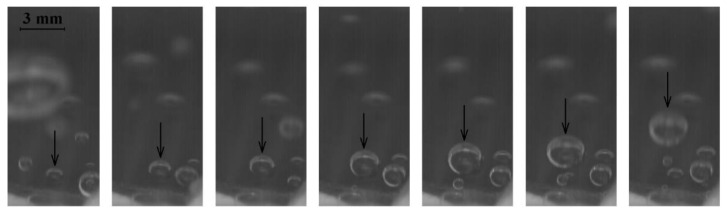
Vapor bubble development sequence (time interval between each photo: 0.04 s) (the arrow marks the same bubble in each photo).

The growth and departure of single vapor bubbles (which merge and form vapor columns at high heat fluxes) from the laser-treated surface might also be an important element that influences the overall boiling performance of the surface. Figure 18 presents a phenomenon of vapor bubble development until its departure. It seems that the bubbles are created at the most favorable locations (e.g., nucleation sites in the form of cavities on the rough laser-made surface) and the new bubble grows at the same location where the previous bubble (that already left the surface) developed—as seen in the figure.

The visual studies can not provide precise information about the boiling performance of the samples in terms of the amount of heat being dissipated from the heaters. In order to obtain detailed data on the thermal performance of each surface, boiling curves need to be drawn based on the temperature readings within the heater and the calculated heat flux values. The detailed procedure for heat flux determination, accuracy analysis, method validation, and the experimental apparatus have been presented by the authors of [42].

**Figure 19:**
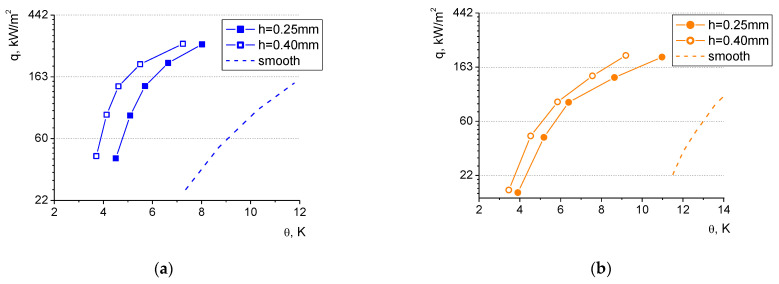
Boiling curves for two microfin heights (0.25 mm and 0.40 mm) for (**a**) water and (**b**) ethyl alcohol.

Figure 19 presents the experimental data of boiling heat flux vs. superheat (defined as the difference between the surface temperature and the saturation temperature of the fluid) for distilled water and ethyl alcohol as working agents considering two microfin heights: 0.25 mm and 0.40 mm (at the same groove width of 1.5 mm and microfin width of 0.5 mm).

The test results clearly show that the increase in the microfin height results in elevated heat flux values, which is undoubtedly related to the extension of the heating surface and the increase in the surface area that dissipates the heat to the liquid. It can also be observed that both of the laser-treated samples significantly outperformed the smooth reference surface (for both the boiling agents). The heat exchange phenomenon on the modified surfaces occurs at much lower superheats and higher heat flux values can be dissipated in comparison to the smooth surface (for example, in the case of water, the heat flux dissipated at the superheat of 8 K from the 0.4 mm height; the laser-treated heater was almost seven times higher than that from the unmodified reference surface). Consequently, laser-made heat exchangers are more efficient. They can effectively work at lower temperature differences which is a considerable advantage for practical applications in the industry (e.g., in heat recovery devices). This favorable phenomenon results from the fact, which has already been already mentioned, that the rough surface provides more nucleation sites of small diameter which become active at lower superheats.

In terms of water, a shift of ca. 1 K towards lower superheats can be observed for the larger microfin height of 0.4 mm in relation to the smaller one of 0.25 mm. For ethanol, this shift is less pronounced and amounts to ca. 0.6 K (on average). This might result from the fact that the surface tension of ethanol is lower, and the influence of the surface morphology might be less visible for this liquid.

**Figure 20:**
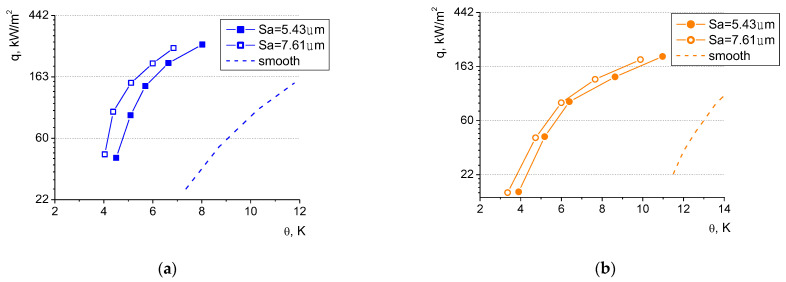
Boiling curves for the same microfin height of 0.25 mm for two surface roughness values for (**a**) water and (**b**) ethyl alcohol.

The discussion of the influence of surface roughness on boiling heat transfer has been focused on the analysis of boiling curves determined for the small microfins (of 0.25 mm groove depth) because, in that case, such an impact will be more clearly visible than for the larger depths. Two values of surface roughness have been considered: 5.43 μm and 7.61 μm. Figure 20 presents the experimental test results for distilled water and ethyl alcohol.

For both the boiling agents, the rougher surface performed better, however, this phenomenon is more clearly visible for water (as seen in Figure 19 with the various heights of the microfins). This further supports the claim that for lower surface tension fluids, the influence of the heater morphology is less pronounced. The boiling curves of the heaters of larger roughness were shifted leftwards to the lower values of superheats by ca. 0.7 K for water and ca. 0.5 for ethanol. Thus, higher heat fluxes can be dissipated to the liquid at the same temperature on the surface. This favorable phenomenon was also observed for higher groove depths, and it was more visible there, probably due to the fact that the surface extension in the ‘macro’ scale—in the form of microfins—proved to be more efficient due to the increased heat exchanging surface area than in the ‘micro’ scale (as the increased surface roughness between the microfins). 

Heat flux values of the laser-treated surfaces presented in Figure 20 (as in Figure 19) can be several times higher than those dissipated from a smooth surface (for both the boiling agents). The difference is particularly evident for low superheats, however, in this region, the comparison might be impossible due to the fact that boiling on the smooth surface is not even initiated at low superheats, and heat transfer occurs via convection without a change of phase. 

**Figure 21:**
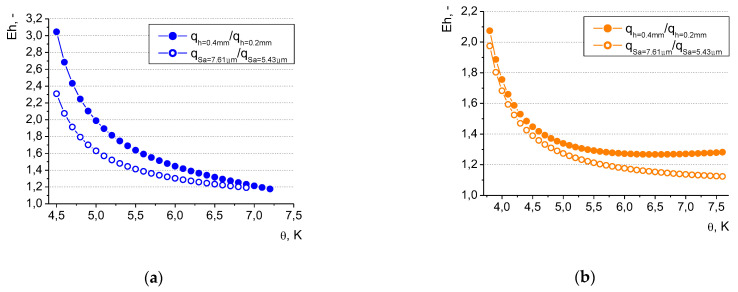
Enhancement ratio for (**a**) water and (**b**) ethyl alcohol.

The details of the thermal performance of higher vs. lower microfins and larger vs. smaller roughness values have been presented in Figure 21 as the enhancement ratio (Eh) —defined as a ratio of the heat flux dissipated from the higher microfin to the heat flux dissipated from the smaller microfin (the same applied to the enhancement ratio determination for surfaces of difference roughness).

The analysis of the above figures indicates that the most significant improvement related to the use of higher microfins and rougher heaters occurs at low superheats. The heat flux dissipated from the longer microfins (of 0.4 mm) was over three times higher in comparison to the heat flux from the microfins of 0.2 mm—for water at the superheat of 4.5 K. In the case of ethanol, lower enhancement ratios were observed (namely, E_h_ = 1.45 for the same superheat of 4.5 K). The influence of surface roughness was less pronounced than that of the microfin height, however of the same nature. At the superheat of 4.5 K and in the case of water, the heat flux dissipated from the rougher surface (Sa = 7.61 μm) was ca. 2.3 times larger than that from the surface of 5.43 μm; while for ethanol, the improvement in the heat flux was smaller—the enhancement ratio amounted to ca. 1.4.

As the surface temperature increases, the advantageous effect of morphology alternation diminishes and stabilizes (however, it is still observed—at least in the analyzed ranges of superheat values—and the enhancement ratio for both the liquids ranges between ca. 1.1 and 1.3). This might be related to a change in the character of the boiling process and its transition from the ‘isolated bubbles’ regime’ into ‘fully developed nucleate boiling’. At large superheats, more and more nucleation sites become active and the surface morphology might play a less decisive role. In terms of the microfin height, in the fully developed mode of boiling, the most important mechanism of heat transfer might be the growth and departure of vapor bubbles (due to the considerable latent heat of vaporization—larger for water than for ethanol, which might also explain the higher enhancement ratio for water) and not convection without phase change (which might increase when the surface area is larger, e.g., for higher microfins).

Experimental analyses are vital for the development of a reliable model of boiling heat transfer on laser-treated surfaces. The test results presented above, and the visual studies presented earlier, have led to the idea of verifying if the Xin and Chao model (originally created for a different type of microstructures) can be successful in determining the heat flux based on the geometrical and material properties of the laser-made heat exchangers.

Xin and Chao [43] developed a boiling heat transfer model for the tunnel microstructures made of longitudinal microfins in the form of ‘T’. It was assumed that this tunnel is filled with vapor, while the vaporization process takes place on its internal surface which is covered with a thin liquid film. The thickness of this film decreases to zero at the heater surface. The authors disregarded the periodic character of bubble growth and departure. They assumed a constant counterflow movement of the vapor phase outside and the liquid phase inside the structure. What is more, a constant temperature value along the height of the tunnel was taken. The experimental constants in the correlation were determined with the regression method (based on the experimental data for water and ethanol) and the following formula for the Nusselt number was given:

(4) Nu=C2h+w2zAr13RemWenPrlp,

where Pr is the Prandtl number, while the Nusselt (Nu), Archimedes (Ar), Reynolds (Re), and Weber (We) numbers are defined as:

(5) $$\mathrm{Nu}=\frac{\alpha s}{\lambda_{l}},$$

(6) $$\mathrm{Ar}=\frac{gz^{3}\left(\rho_{l}-\rho_{v}\right)}{\nu_{l}^{2}\rho_{l}},$$

(7) Re=2qsrμl,

(8) $$\mathrm{We}=\frac{q^{2}s^{2}}{\sigma \rho_{v}r^{2}z},$$

**Figure 22:**
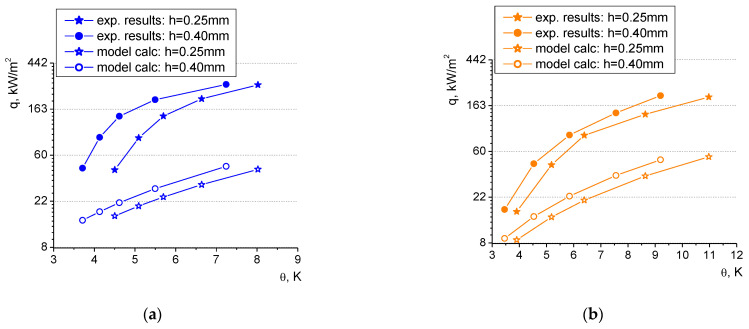
Comparison of the experimental and calculated results for (**a**) water and (**b**) ethyl alcohol.

The constants’ values are as follows: C = 3.76, m = −0.15, *n* = 0.29, and *p* = 0.76. The geometrical parameters of the samples are expressed as s—width of a single cell in the tunnel, z—width of the tunnel opening, w—tunnel width, and h—height. The comparison of the results obtained according to the Xin and Chao model with the experimental data of the laser-treated samples is presented in Figure 22. The necessary modifications (in order to perform the calculations) consisted of assuming z = w (due to the fact that the laser-made grooves are fully open to the liquid) and s = w + a (according to Figure 1a).

As can be seen, the model underestimates the values of heat flux (especially in the case of water). For example, the actual values of water boiling heat flux recorded at the superheat of 5.5 K during measurements on surfaces covered with 0.4 mm and 0.25 mm microfins were, respectively, 6.6 and 5.3 times higher than the calculation results obtained with the model. In the case of ethanol and the same superheat of 5.5 K, the discrepancies were lower and the respective values were 3.7 and 3.4. Generally, in the whole range of superheats, the experimental data were higher than the results of model calculations by 2.7–6.9 times for water and by 1.9–4.0 times for ethanol.

**Figure 23:**
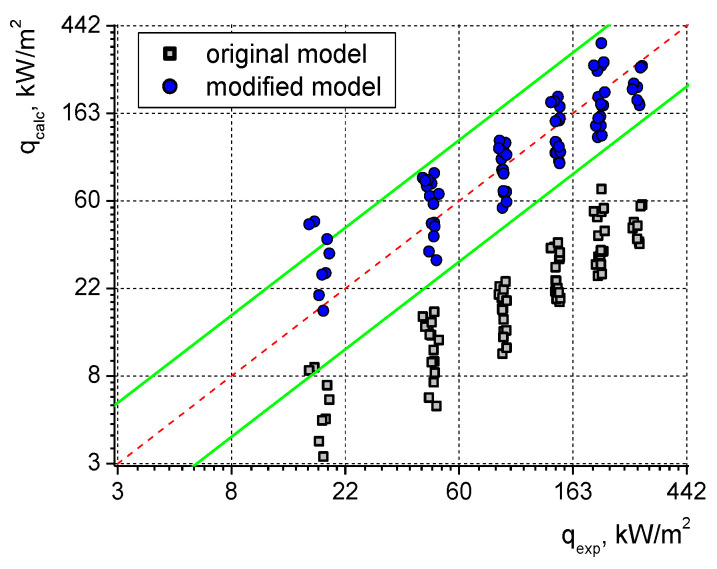
Experimental vs. model calculation results for the original and modified correlations; dashed red line: q_calc_ = q_exp_ and solid green lines: ±100% agreement bands.

However, the model provides quite consistent results and the nature of heat flux changes is quite well-reflected. Thus, a modification to this model has been proposed. Namely, due to a different geometry of the laser-made grooves, “z” will equal “w” (the laser grooves are fully open to the liquid as opposed to the original model, in which the gap at the top was small due to the microfins’ shape in the form of a “T”) and s = w + a. Moreover, the values of two constants in Equation (4) have been determined for the laser-processed samples: C (which would consider the unique feature of surface roughness at the bottom of the laser-treated samples) as well as constant p (which is related to the properties of a given liquid and how it interacts with a laser processed surface; Figure 22 indicates the influence of the liquid used for heat flux determination). Using the fitting procedure for the experimental results of 80 data points (taken from the current paper and [42]), the following constants have been found for the laser-processed surfaces: C = 9.11 and *p* = 0.79. Figure 23 presents the comparison of the experimental and calculated values for the original and modified model. The red dashed lines indicate full agreement with the model (q_calc_ = q_exp_), while the green lines are ±100% agreement bands. The original model provided weak agreement with the model (as was also indicated in Figure 22), while in the case of the modified correlation, almost all data points are within the ±100% agreement bands.

It needs to be added that the above modification of the model originally proposed by Xin and Chao (for a different type of microstructure) provides higher accuracy than a modified correlation developed by the authors, based on the concept of a matrix of equivalent microfins (as originally presented by Smirnov [44]). Thus, it seems that the convection-based model of boiling heat transfer might be more applicable to laser-treated surfaces. 

## 4. Conclusions

Laser treatment technology enables the modification of the morphology of surfaces in a number of ways. The impact of the laser beam on the heater can be very advantageous and lead to the improvement of heat transfer conditions in the nucleate boiling mode. The following conclusions can therefore be drawn based on the presented data:

1. The depth of the laser grooves is proportional to the pulse duration and inversely proportional to the scanning velocity. Both of these phenomena are interconnected and might be related to the interaction time of the laser beam with the surface. The largest value of the groove depth (microfin height) in the present study was reported for a pulse duration value of 250 ns and a scanning velocity of 200 mm/s.
2. Surface roughness increases with the rise in pulse duration. It also typically increases with a lower scanning velocity, however, this impact was clearly visible only for the lowest pulse duration of 65 ns. The roughest surface at the bottom of the grooves was obtained with a pulse duration value of 250 ns and a scanning velocity of 200 mm/s.
3. Laser-treated samples provided significant enhancement of boiling heat transfer compared with a smooth reference surface. This might be linked to both surface extension (in the form of microfins) and increased surface roughness, which can considerably improve heat transfer conditions—especially in the range of small superheats.
4. The surface with higher microfins (larger grooves) outperformed that with lower ones, especially at small superheats, while at higher temperature differences the discrepancies became less visible. The same phenomenon has been observed for surface roughness. These results might be linked with the surface extension provided by the longer microfins and larger nucleation sites’ density for the rougher sample. The impact of these two factors seems to be most pronounced for small heat flux values, wherein the heat flux dissipated from the sample containing microfins of 0.4 mm height was over three times (for water) and two times (for ethanol) higher than for the sample with smaller microfins (0.2 mm high). The influence of surface roughness (considering the two values of this parameter: 5.43 μm and 7.61 μm) was slightly less pronounced (especially in the case of water) but of the same nature.
5. A modified correlation for the laser-processed surfaces, based on the model by Xin and Chao, was proposed and compared with the original correlation. A rather satisfactory agreement between the experimental and calculated results was observed for the modified model for two boiling agents (water and ethanol).

## Figures and Tables

**Table 1:** Details of the geometrical parameters of the circular samples.

Groove Depth (Fin Height) h, mm	Groove Width w, mm	Fin Width a, mm
Boiling tests and correlation development:		
0.40	1.50	0.50
0.25	1.50	0.50
Correlation development:		
0.55	1.15	0.50
0.25	1.15	0.50
0.55	1.15	1.10
0.25	1.15	1.10
0.55	0.60	1.10
0.25	0.60	1.10

## Data Availability

All data available upon request from the authors.

## Nomenclature

α	heat transfer coefficient, W/(m^2^K)
λ	thermal conductivity, W/(mK)
μ	dynamic viscosity, Ns/m^2^
ν	kinematic viscosity, m^2^/s
θ	superheat, K
ρ	density, kg/m^3^
σ	surface tension, N/m
a	fin width, m
Ar	Archimedes number
C	constant, -
Cu	copper
Eh	enhancement ratio
g	gravitational acceleration, m/s^2^
h	height/groove depth, m
l	liquid
L	distance, m
n	constant, -
Nu	Nusselt number
p	constant, -
Pr	Prandtl number
q	heat flux, W/m^2^
r	heat of vaporisation, J/kg
Re	Reynolds number
s	width of a single cell in the tunnel, m
Sa	surface roughness, µm
sat	value at the saturated state
t	pulse duration
T	temperature, K
w	tunnel width, m
v	vapor; scanning velocity, mm/s
z	width of the tunnel opening, m.
